# Adverse events in the treatment of spinal muscular atrophy in children and adolescents with nusinersen: A systematic review and meta-analysis

**DOI:** 10.3389/fped.2023.1152318

**Published:** 2023-04-25

**Authors:** Zhi-Juan Zhong, Pi-Mei Zheng, Hui-Hong Dou, Ji-Gan Wang

**Affiliations:** ^1^ Department of Pediatrics, Hainan General Hospital, Hainan Affiliated Hospital of Hainan Medical University, Haikou, China; ^2^ Department of Pediatrics, Guangxi Clinical Research Center for Pediatric Diseases, Maternal and Child Health Hospital of Guangxi Zhuang Autonomous Region, Nanning, China

**Keywords:** spinal muscular atrophy, nusinersen, SMA, meta, AES

## Abstract

**Objectives:**

To systematically analyze adverse events (AEs) in treatment of spinal muscular atrophy (SMA) with Nusinersen in children and adolescents.

**Methods:**

The study is registered on PROSPERO (CRD42022345589). Databases were searched and literature relating to Nusinersen in the treatment of spinal muscular atrophy in children from the start of database establishment to December 1, 2022, was retrospectively analyzed. R.3.6.3 statistical software was used, and random effects meta-analysis was performed to calculate weighted mean prevalence and 95% confidence intervals (CI).

**Results:**

In total, 15 eligible studies were included, with a total of 967 children. Rate of definite Nusinersen-related AEs was 0.57% (95% CI: 0%–3.97%), and probable Nusinersen-related AEs 7.76% (95% CI: 1.85%–17.22%). Overall rate of AEs was 83.51% (95% CI: 73.55%–93.46%), and serious AEs 33.04% (95% CI: 18.15%–49.91%). For main specific AEs, fever was most common, 40.07% (95% CI: 25.14%–56.02%), followed by upper respiratory tract infection 39.94% (95% CI: 29.43%–50.94%), and pneumonia 26.62% (95% CI: 17.99%–36.25%).

The difference in overall AE rates between the two groups (Nusinersen group and placebo group) was significant (OR = 0.27,95% CI: 0.08–0.95, *P *= 0.042). Moreover, incidence of serious adverse events, and fatal adverse events were both significantly lower than in the placebo group (OR = 0.47, 95%CI: 0.32–0.69, *P *< 0.01), and (OR = 0.37, 95%CI: 0.23–0.59, *P *< 0.01), respectively.

**Conclusion:**

Nusinersen direct adverse events are rare, and it can effectively reduces common, serious, and fatal adverse events in children and adolescents with spinal muscular atrophy.

## 1. Introduction

Spinal muscular atrophy (SMA) is a progressive, autosomal recessive, motor neuron disease, which is caused by mutations on chromosome 5 of the SMN1 gene (1). The main clinical features of patients were progressive muscle weakness and muscle atrophy resulting from the degeneration of motor neurons in the anterior horn of spinal cord (2). The prevalence of SMA is 1–2 in 100,000 in developing countries (3). Furthermore, SMA is the most fatal genetic disease in children under 2 years of age; SMA affects the respiratory system, with respiratory failure being the most common cause of death (4). Nusinersen was the first approved disease-modifying treatment for SMA. It is an antisense oligonucleotide drug, which modifies pre-mRNA splicing to promote inclusion of SMN2 exon 7, which increases production of full-length SMN proteins (5). In clinical trials, Nusinersen was demonstrated to significantly improve both motor function and overall survival (6). As Nusinersen is unable to penetrate the blood-brain barrier, Nusinerson needs to be administered by repeat intrathecal injection at an injection dose of 12 mg (5 ml) each time. Typically, the first three doses are given 14 days apart, and a fourth dose given 30 days after the third dose. After this, maintenance therapy should be administered every 4 months, usually for more than 5 years (7). Platelet count, prothrombin time (PT), activated partial thromboplastin time (APTT) and urine protein should be measured before administration. Common adverse events of Nusinersen include respiratory infections and constipation, though some patients develop thrombocytopenia and coagulopathy, increasing their risk of nephrotoxicity (8). Nusinersen is proven to have significant clinical benefits for SMA in children, improving motor function.

Clinical use of a drug is associated with not only curative effect, but also the safety of the drug, which affects its clinical application. However, till date, there are no meta-analysis studies on the adverse events of nociception in children and adolescents. Therefore, this study used meta-analysis to systematically review existing published studies on adverse events during the treatment of SMA with Nusinersen in children and provide high-quality evidence for clinical practice.

## 2. Methods

The protocol of this systematic review and meta-analysis has been registered with the international prospective register of systematic reviews (PROSPERO) platform, following the PRISMA-P guidelines. The protocol is assigned the systematic review registration number, PROSPERO CRD42022345589, available from https://www.crd.york.ac.uk/PROSPERO/display_record.php?ID=CRD42022345589.

### 2.1. Search strategy and data extraction

PubMed, Web of Science, EMBASE, Medline, and Cochrane Library Databases were searched to collect literature relating to the use of Nusinersen for treatment of children with SMA. The search time limit was from the establishment of the database to December 1, 2022. At the same time as the online database and manual search were performed, the references of included literature were traced back. Both keywords and free text were used in the retrieval, and adjusted according to the different database characteristics. The search criteria for retrieval were as follows, consistently search for “Paediatrics” OR “Pediatrics” AND (Nusinersen) OR (SPINRAZA) AND (Spinal Muscular Atrophy) OR (SMA).

### 2.2. Literature screening and inclusion/exclusion criteria

In total, two researchers independently searched and screened the literature, collected data, and cross-checked. In the event of a dispute, a consensus was reached through discussion or negotiation with a further researcher. The inclusion criteria were defined firstly by study type, cohort study, case-control study, or case series analysis. Secondly, by research object, children who underwent Nusinersen treatment. Finally, by observation indicators, the number of samples and adverse events the study reported. Exclusion criteria included case reports, adult research, and either incomplete or missing data or an inability to obtain data from the study author.

### 2.3. Adverse event grade assessment

Adverse events were graded as mild or moderate (grades 1–2), and serious (grades 3–4) according to the Common Terminology Criteria for Adverse Events v5.0 (CTCAE v5.0) (U.S. Department of Health and Human Services; National Institutes of Health; National Cancer Institute, 2017) (9).

### 2.4. Literature quality evaluation

Quality assessment of the included studies was performed using different tools dependent on the study design. Randomized controlled trials were assessed following Cochrane collaboration handbook version 5.1.0. Case series quality assessments were performed following the IHE (Institute of Health Economics) quality appraisal checklist (Institute of Health Economics (IHE) (10).

### 2.5. Statistical analysis

R 3.6.3 statistical software was used for meta-analysis. Continuous variables were described by weight mean difference (WMD) and the 95% confidence interval (CI). Prior to meta-analysis, a normality test was performed to determine both before the original and the converted rate. The effect rate sizes for each independent sample were pooled to calculate both the incidence and 95% CI. Heterogeneity between the included studies was assessed by *Q*-test and *I*^2^ statistics. Dichotomous variables were described by relative risk (RR) and its 95% CI. In cases where there was no statistical heterogeneity among the results (*P* > 0.1 and *I*^2^ < 50%), the fixed-effects model was used for meta-analysis. Conversely, in cases indicative of statistical heterogeneity, the random-effects model was used for Meta-analysis (*α* = 0.05). Egger's method was then used to statistically test the publication deviation.

## 3. Results

### 3.1. Literature search

**Figure 1:**
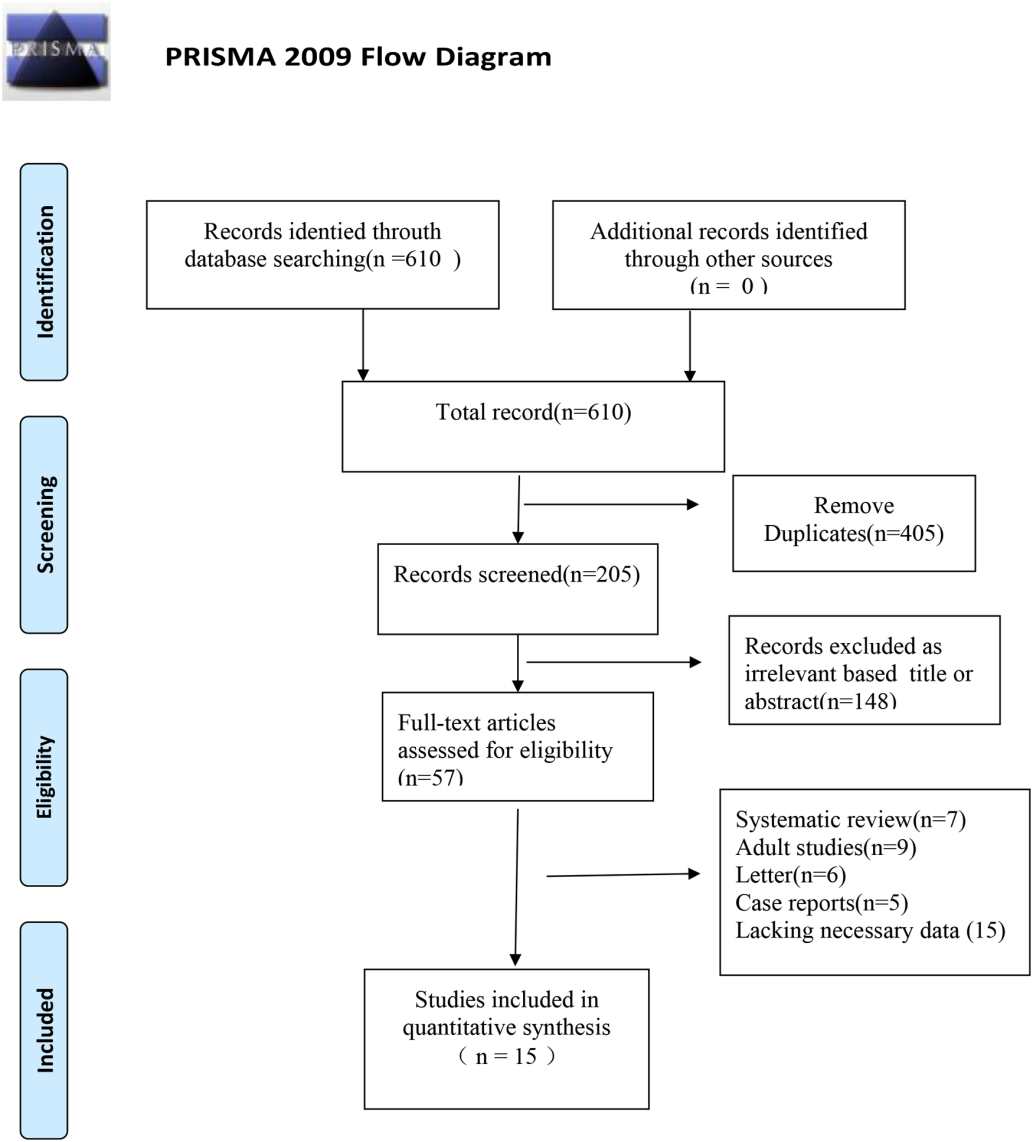
Flow diagram for identification of selected studies in the meta-analysis.

In total, 610 articles were retrieved from the literature databases. Endnote X9 was used to remove duplicate articles leaving 205 articles. After reading the titles and abstracts, 148 non-conforming articles were excluded, and 57 articles were included for double screening. After reading the full text, 15 articles (11–25) were included in the final review. The process and results of literature screening are shown in Figure 1.

### 3.2. Basic characteristics and quality evaluation of the study

**Figure 2:**
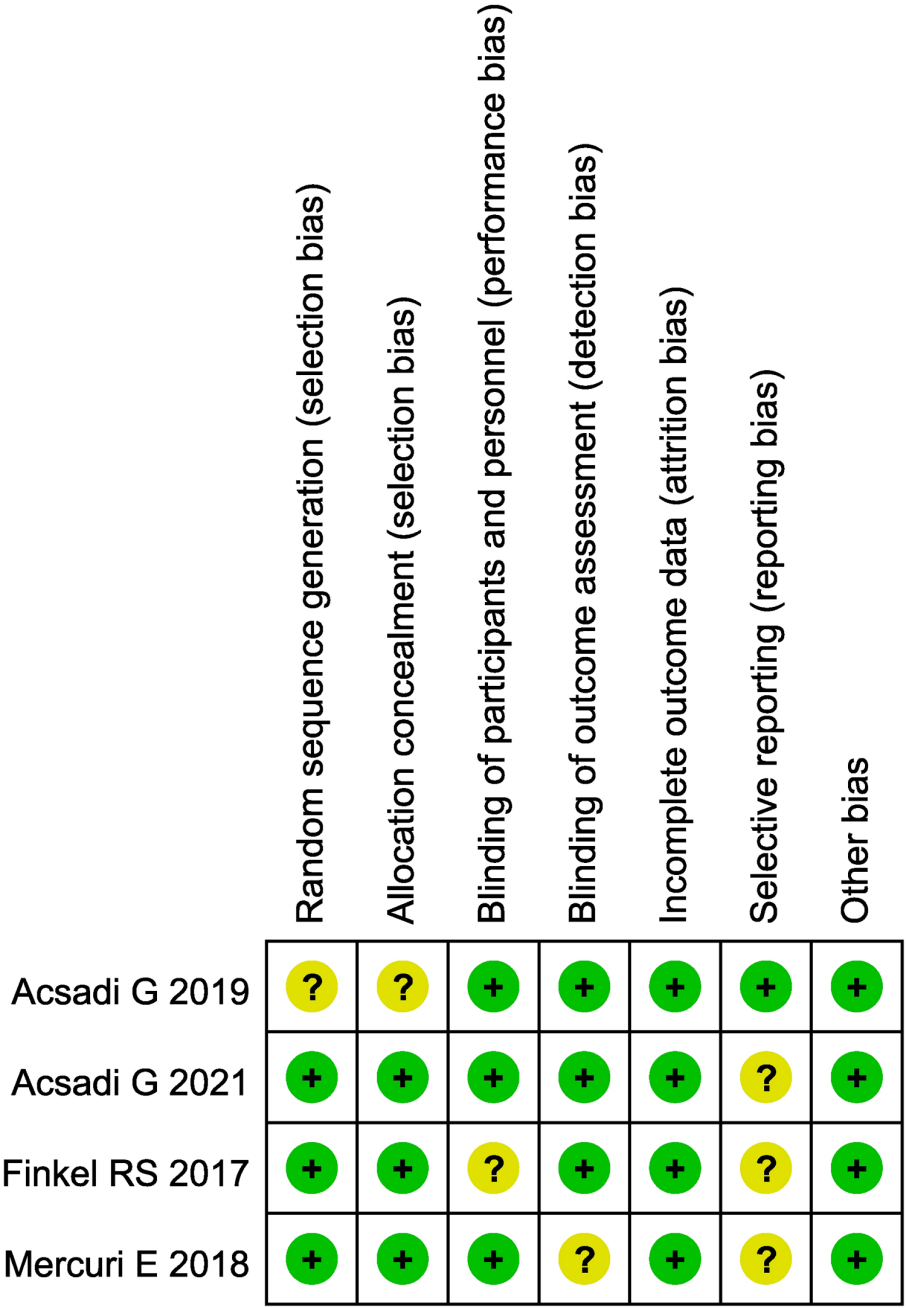
Evaluation of the quality of included randomized controlled trials.

The basic characteristics and quality evaluation of the study included 15 articles in English, totaling 967 patients. There were 4 articles which were randomized controlled trials (RCT), the remaining 11 were case series. The basic characteristics of the included studies are shown in Table 1. Evaluation of the literature quality of the 4 RCTs is shown in Figure 2, and the 11 case series in Table 2.

**Table 1:** Characteristic of included stduies.

study	Total patients	Serious adverse events	The overall rate of adverse events	Nusinersen-related adverse events	May be related to drugs	Upper respiratory tract infection	Pneumonia	Vomiting	Constipation	Diarrhea	Feve	Pain	Cough	Respiratory failur	Gastroesophageal reflux disease	Atelectasis	Dysphagia	Aspiration pneumonia	Respiratory distress	Nasopharyngitis	Skin rash	Otitis media	Thrombocytopenia	Post–lumbar Puncture Syndrome (PLPS)
Finkel RS[11]2017	80	45	77	N	N	24	23	14	28	N	45	N	14	20	10	18	8	8	21	N	N	N	N	N
Konersman CG[12]2021	19	N	N	N	N	2	3	4	0	5	5	4	3	3	0	0	1	1	2	0	0	1	0	N
De Vivo DC[13]2019	25	12	25	0	8	19	6	10	6	7	21	N	13	N	N	N	N	N	N	7	N	7	0	8
Darras BT[14]2019	240	99	231	1	41	90	40	58	49	N	114	47	43	28	N	25	N	N	34	59	N	N	38	27
Acsadi G[15]2021	20	10	20	0	2	12	13	10	4	6	16	8	14	4	N	N	N	N	4	N	N	N	1	N
Szabó L[16]2020	54	N	N	0	1	N	N	N	N	N	N	N	N	N	N	N	N	N	N	N	N	N	1	3
Pechmann A[17]2018	61	29	53	N	N	31	N	N	N	N	N	N	N	8	N	N	N	N	N	N	3	N	N	3
Audic F[18]2020	25	N	N	N	N	N	N	4	N	N	1	2	N	N	N	N	N	N	N	N	N	N	N	6
Chiriboga CA[19]2016	28	5	25	0	2	5	N	3	3	N	4	7	N	N	3	N	N	N	N	N	N	N	N	12
Mercuri E[20]2018	84	14	78	N	N	25	N	24	N	N	36	45	21	N	N	N	N	N	N	24	0	0	0	8
Osredkar D[21]2021	61	0	24	13	13	N	N	N	N	N	N	N	N	N	N	N	N	N	N	N	N	N	N	10
Finkel RS[22]2021	20	16	20	0	0	12	8	8	9	N	17	4	9	8	6	11	N	N	N	8	12	8	N	N
Haché M[23]2016	28	N	20	N	N	N	N	1	N	N	N	N	N	N	N	N	N	N	N	N	N	N	N	9
Sansone VA[24]2019	50	N	N	0	0	N	N	3	N	N	3	N	N	N	N	N	N	N	N	N	N	N	N	3
Wataya T[25]2021	172	23	67	N	N	N	30	N	N	N	34	25	N	N	N	N	N	N	N	N	N	N	N	12

N, not mentioned.

**Table 2:** Quality assessment and risk of bias of included case series (IHE).

Study	Study objective	Study design	Study population	Intervention and co-intervention	Outcome measures	Statistical analysis	Results and conclusions	Competing interests and sources of support	Total
Konersman CG2021	1/1	1/3	3/3	2/2	3/4	1/1	4/5	1/1	16/20
De Vivo DC2019	1/1	1/3	3/3	2/2	3/4	1/1	4/5	1/1	16/20
Szabó L2020	1/1	2/3	3/3	2/2	3/4	1/1	4/5	1/1	17/20
Pechmann A2018	1/1	2/3	3/3	2/2	3/4	1/1	4/5	1/1	17/20
Audic F2020	1/1	2/3	3/3	2/2	3/4	1/1	4/5	1/1	17/20
Chiriboga CA2016	1/1	2/3	3/3	2/2	3/4	1/1	4/5	1/1	17/20
Osredkar D2021	1/1	1/3	3/3	2/2	2/4	1/1	4/5	1/1	15/20
Finkel RS2021	1/1	1/3	3/3	2/2	2/4	1/1	4/5	1/1	15/20
Haché M2016	1/1	1/3	3/3	2/2	2/4	1/1	3/5	1/1	14/20
Sansone VA2019	1/1	1/3	3/3	2/2	2/4	1/1	4/5	1/1	15/20
Wataya T2021	1/1	1/3	2/3	2/2	2/4	1/1	4/5	1/1	14/20

### 3.3. Results of meta-analysis

**Figure 3:**
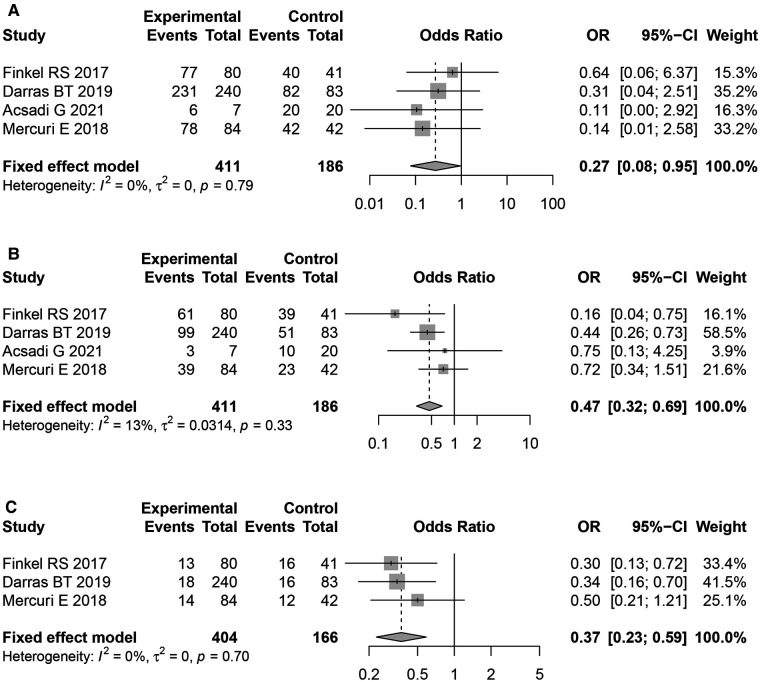
Forest plot of AEs in nusinersenand control groups.

All the included literature studies were in English. Use of Nusinersen in the treatment of children with SMA had a motor milestone change of 61.38% (95%CI: 37.60%–82.58%). Most of the adverse events which occurred during Nusinersen treatment were related to the primary SMA disease and its related complications. The rate of definite Nusinersen-related adverse events was 0.57% (95% CI: 0%–3.97%), whilst the rate of probable Nusinersen-related adverse events was 7.76% (95% CI: 1.85%–17.22%). Moreover, the overall rate of adverse events was 83.51% (95% CI: 73.55%–93.46%), whilst the rate of serious adverse events was 33.04% (95% CI: 18.15%–49.91%). Among specific adverse events, fever was most common 40.07% (95% CI: 25.14%–56.02%), followed by upper respiratory tract infection 39.94% (95% CI: 29.43%–50.94%). Other specific adverse events included pneumonia 26.62% (95% CI: 17.99%–36.25%), pain 22.75% (95% CL: 15.25%–31.25%), respiratory failure 18.77% (95% CI: 11.9%–26.77%), respiratory distress 17.83% (95% CI: 11.13%–25.71%), aspiration pneumonia 17.83% (95% CI: 11.13%–25.71%), vomiting 21.4%, constipation 19.59%, diarrhea 28.11%, cough 31.81%, acid gastroesophageal reflux disease 10.08%, atelectasis 16.2%, dysphagia 8.98%, nasopharyngitis 21.19%, skin rash 7.16%, otitis media 12.56%, thrombocytopenia 1.81%, and post–lumbar puncture syndrome(PLPS) 14.16% (Table 3). Furthermore, a statistically significant difference in overall adverse event rates between the Nusinersen group and the placebo control group was observed [OR = 0.27,95% CI: 0.08–0.95, *P *= 0.042 (Figure 3A). The incidence of serious adverse events [OR = 0.47, 95%CI: 0.32–0.69, *P *< 0.01 (Figure 3B)], and fatal adverse events [OR = 0.47, 95%CI: 0.32–0.69, *P *< 0.01 (Figure 3B)], [OR = 0.37, 95%CI: 0.23–0.59, *P *< 0.01 (Figure 3C)] in the Nusinersen group were also significantly lower than in the placebo group.

**Table 3:** Meta-analysis results.

AEs	Number of included studies	Sample size	*P*	*I* ^2^	*t* ^2^	*Effect of model*	R% (95%CI)
Overall rate of AEs	11	819	<0.01	97%	0.026	Random	83.51% (95% CI: 73.55%–93.46%)
Serious AEs	10	791	<0.01	95%	0.071	Random	33.04% (18.15%–49.91%)
Definite Nusinersen-related AEs	8	498	<0.01	84	0.026	Random	0.57% (0–3.97%)
Probable Nusinersen-related AEs	8	498	<0.01	88	0.038	Random	7.76% (1.85%–17.22%)
Upper respiratory tract infection	9	577	<0.01	83	0.022	Random	39.94% (29.43%–50.94%)
Pneumonia	7	576	<0.01	79	0.013	Random	26.62% (17.99% –36.25%)
Vomiting	11	619	<0.01	76	0.016	Random	21.04% (14.15%–28.89%)
Constipation	9	482	<0.01	82	0.02	Random	19.59% (10.23%–31.09%)
Diarrhea	3	64	0.97	0	0	Fixed	28.11% (17.86%–39.67%)
Fever	11	763	<0.01	94	0.066	Random	40.07% (25.14%–56.02%)
Pain	8	608	<0.01	76	0.013	Random	22.75% (15.25%–31.25%)
Cough	7	488	<0.01	85	0.024	Random	31.81% (20.32%–44.57%)
Respiratory failure	6	440	0	65	0.08	Random	18.77% (11.9%–26.77%)
Gastroesophageal reflux disease	4	142	<0.01	78	0.028	Random	10.08% (1.78%–24.02%)
Atelectasis	4	399	<0.01	91	0.043	Random	16.2% (3.71%–35.17%)
Dysphagia	2	99	0.48	0	0	Fixed	8.98% (4.18%–15.37%)
Aspiration pneumonia	4	189	<0.01	0	0	Fixed	17.83% (11.13%–25.71%)
Respiratory distress	4	359	0.1	53	0.05	Random	17.83% (11.13%–25.71)
Nasopharyngitis	5	388	<0.01	83	0.022	Random	21.19% (10.38%–34.58%)
Skin rash	4	194	<0.01	94	0.104	Random	7.16% (0–31.82%)
Otitis media	4	148	<0.01	93	0.115	Random	12.56% (0.03%–42.34%)
Thrombocytopenia	6	442	<0.01	92	0.047	Random	1.81% (0–9.95%)
Post–lumbar puncture syndrome	11	828	<0.01	78	0.01	Random	14.16% (9.13%–20.1%)
motor milestone change	7	350	<0.01	95	0.098	Random	61.38% (37.60%–82.58%)

AEs, adverse events.

### 3.4. Subgroup analysis

**Figure 4:**
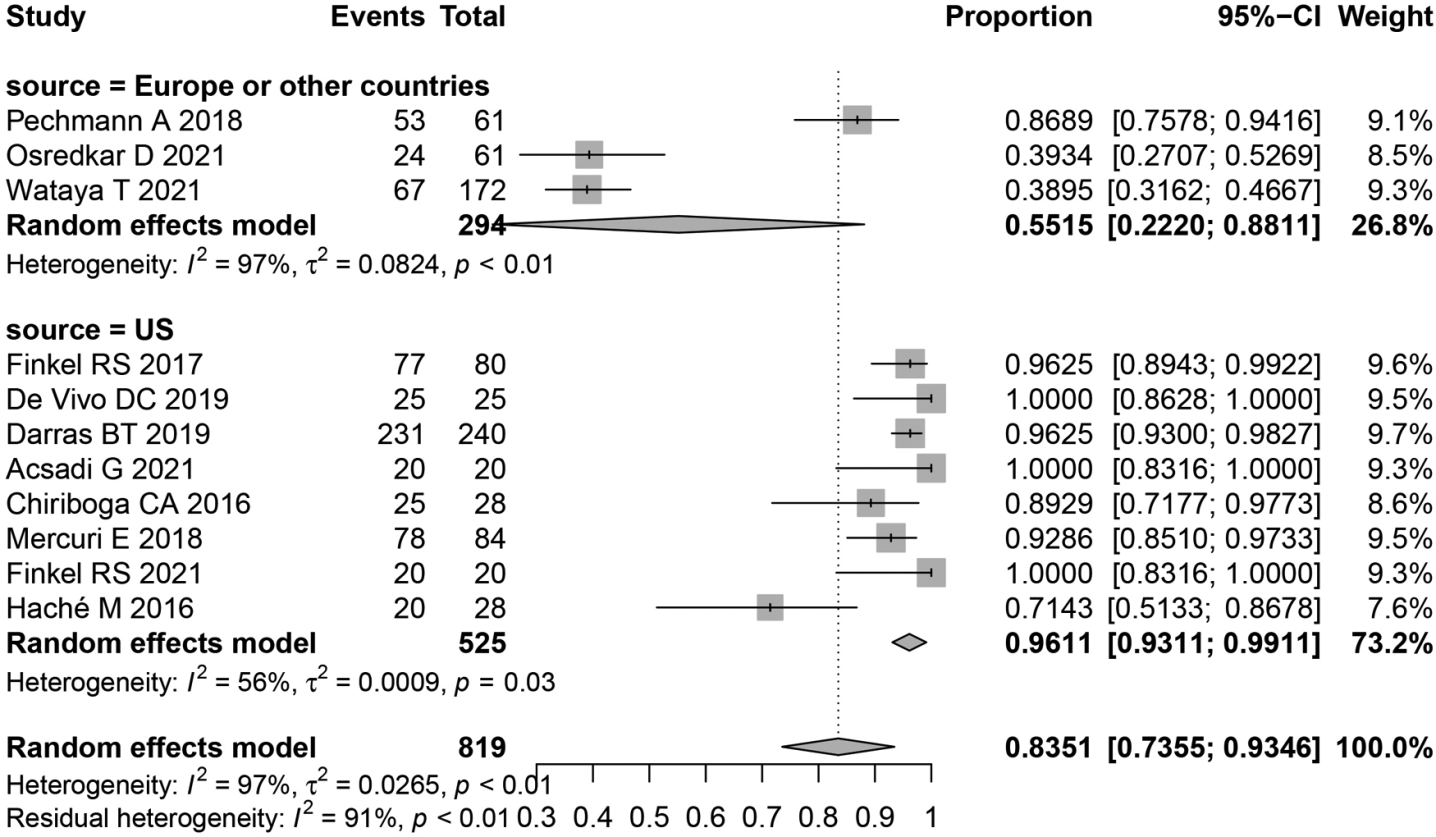
Forest plot of AEs subgroup analysis.

Most adverse events in this study were of high heterogeneity. Thus to determine the source of heterogeneity a subgroup analysis of overall adverse events was performed, subgroups included, United States, Europe, and other countries. The results following subgroup analysis were consistent with the overall results, indicating no significant differences between heterogeneity and overall heterogeneity in each subgroup. This suggests that the region studied was not the main source of heterogeneity (Figure 4).

## 4. Discussion

With the development and progression of SMA, muscle weakness can lead to abnormalities of the skeletal, respiratory, and digestive systems. Respiratory failure is the most common cause of death relating to SMA. For decades, treatment options for SMA have been limited to supportive physical, respiratory, and orthopedic corrective therapies, which aim to alleviate symptoms and improve residual function. Nusinersen was first approved by the U.S. Food and Drug Administration (FDA) in December 2016, as the first approved disease-modifying treatment for SMA, providing a new treatment hope (26). Nusinersen is an antisense oligonucleotide drug, which exerts its therapeutic effect through modification of pre-mRNA splicing to promote inclusion of SMN2 exon 7. This increases production of full-length SMN protein (27). Whilst Nusinersen has been shown to significantly improve motor function and overall survival, to date, it has not been curative, being effective only in relieving symptoms.

There have been no large-sample RCT studies of adverse events in pediatric patients with SMA treated with Nusinersen. In particular, long-term follow-up data of adverse events is lacking. In this meta-analysis study, the overall motor milestone change and adverse event rate in the treatment of SMA with Nusinersen were 61.38% and 83.51%, respectively. However, most adverse events were related to the primary SMA disease. In contrast, the rate of definite Nusinersen drug-related adverse events was 0.57% (95% CI: 0%–3.97%), and the rate of probable Nusinersen drug-related adverse events was 7.76% (95% CI: 1.85%–17.22%). The Darras BT (14) report showed that only 1 of the 240 patients with vomiting had a clear association with Nusinersen, whilst it was possible that Nusinersen caused headache (*n* = 9), fever *(n* = 8), back pain (*n* = 7), PLPS (*n* = 4), vomiting (*n* = 3) and tachycardia (*n *= 2). However, possibly, these effects could have been caused by the lumbar puncture procedure rather than Nusinersen. In a further study (14), 38 cases of thrombocytopenia were reported, though there were also thrombocytopenia cases in the SMA control group that did not receive Nusinersen. Therefore, it was not possible to assess whether thrombocytopenia was Nusinersen drug-induced or caused by SMA disease itself. However, Szab ól (16) reported 1 case of possible drug-induced thrombocytopenia. Moreover, Osredkar D (21) reported 13 cases of drug-related adverse reactions; however, all of them were mild adverse events, the majority of which occurred in the load stage of Nusinersen treatment.

Although most SMA patients treated with Nusinersen reported improvements in motor function, these improvements were not significant across all age groups. Moreover, whilst Nusinersin has received full contingent approval from the US FDA and the European Medicines Agency, Nusinersen is yet to be approved by other regulatory agencies in other countries, particularly in low and middle-income countries (28).

Nusinersen cannot cross the blood-brain barrier; therefore, Nusinersen is administered intrathecally. This presents challenges for pediatric patients with SMA, such as the need for repeated anesthesia/sedation. Especially in infants and young children, intrathecal administration increases anesthesia-related risks and the cost of surgical management. Moreover, intrathecal injection is associated with lower back pain, dizziness, and nausea. In this study, 14.16% of the PLPS were caused by the lumbar puncture procedure, rather than Nusinersen itself.

This review does have limitations. Firstly, there is a lack of long-term follow-up data of adverse events in children with SMA treated with Nusinersen, with the drug not having been officially approved for use in China until October 2019. Secondly, most of the included studies were case series with only small sample sizes. Different trial protocols may affect the true representation of the study population, thereby limiting the conclusions drawn in this review. Thirdly, there is large heterogeneity in the studies included in this meta-analysis, which may affect the accuracy of the results.

## 5. Conclusion

Currently, there is no cure for SMA; however, Nusinersen is the first choice and an effective and safe drug for SMA management, causing few directly drug-related adverse events. Moreover, Nusinersen can effectively reduce the incidence of general, serious, and fatal adverse events in SMA and its associated complications in children. This suggests that Nusinersen is worthy of continuation in SMA treatment, and further clinical investigation is warranted to maximize therapeutic benefits.
